# Parent proxy-report of their children's health-related quality of life: an analysis of 13,878 parents' reliability and validity across age subgroups using the PedsQL™ 4.0 Generic Core Scales

**DOI:** 10.1186/1477-7525-5-2

**Published:** 2007-01-03

**Authors:** James W Varni, Christine A Limbers, Tasha M Burwinkle

**Affiliations:** 1 Department of Pediatrics, College of Medicine, Department of Landscape Architecture and Urban Planning, College of Architecture, Texas A&M University, 3137 TAMU, College Station, TX 77843-3137, USA; 2 Department of Psychology, College of Liberal Arts, Texas A&M University, College Station, TX 77843-3137, USA; 3 The Children's Hospital at Scott & White, Department of Pediatrics, College of Medicine, Texas A&M University Health Science Center, 2401 South 31^st ^Street, Temple, TX 76508, USA

## Abstract

**Background:**

Health-related quality of life (HRQOL) measurement has emerged as an important health outcome in clinical trials, clinical practice improvement strategies, and healthcare services research and evaluation. While pediatric patient self-report should be considered the standard for measuring perceived HRQOL, there are circumstances when children are too young, too cognitively impaired, too ill or fatigued to complete a HRQOL instrument, and reliable and valid parent proxy-report instruments are needed in such cases. Further, it is typically parents' perceptions of their children's HRQOL that influences healthcare utilization. Data from the PedsQL™ Database^SM ^were utilized to test the reliability and validity of parent proxy-report at the individual age subgroup level for ages 2–16 years as recommended by recent FDA guidelines.

**Methods:**

The sample analyzed represents parent proxy-report age data on 13,878 children ages 2 to 16 years from the PedsQL™ 4.0 Generic Core Scales Database^SM^. Parents were recruited from general pediatric clinics, subspecialty clinics, and hospitals in which their children were being seen for well-child checks, mild acute illness, or chronic illness care (n = 3,718, 26.8%), and from a State Children's Health Insurance Program (SCHIP) in California (n = 10,160, 73.2%).

**Results:**

The percentage of missing item responses for the parent proxy-report sample as a whole was 2.1%, supporting feasibility. The majority of the parent proxy-report scales across the age subgroups exceeded the minimum internal consistency reliability standard of 0.70 required for group comparisons, while the Total Scale Scores across the age subgroups approached or exceeded the reliability criterion of 0.90 recommended for analyzing individual patient scale scores. Construct validity was demonstrated utilizing the known groups approach. For each PedsQL™ scale and summary score, across age subgroups, healthy children demonstrated a statistically significant difference in HRQOL (better HRQOL) than children with a known chronic health condition, with most effect sizes in the medium to large effect size range.

**Conclusion:**

The results demonstrate the feasibility, reliability, and validity of parent proxy-report at the individual age subgroup for ages 2–16 years. These analyses are consistent with recent FDA guidelines which require instrument development and validation testing for children and adolescents within fairly narrow age groupings and which determine the lower age limit at which reliable and valid responses across age categories are achievable. Even as pediatric patient self-report is advocated, there remains a fundamental role for parent proxy-report in pediatric clinical trials and health services research.

## Background

Health-related quality of life (HRQOL) measurement has emerged as an important health outcome in clinical trials, clinical practice improvement strategies, and healthcare services research and evaluation [1,2]. A HRQOL instrument must be multidimensional, consisting at the minimum of the physical, psychological (including emotional and cognitive), and social health dimensions delineated by the World Health Organization [3,4]. During the past several years, legislative changes have created both voluntary and mandatory guidelines for drug studies in children, resulting in a substantial increase in pediatric clinical trials. Recently, the U.S. Food and Drug Administration (FDA) published draft guidance for industry in which the FDA describes how it evaluates patient-reported outcome (PRO) instruments as efficacy outcomes in clinical trials [4]. In this draft document, the FDA articulated the importance of *patient-reported *outcomes in clinical trials, including those for pediatric patients [4].

It is well documented in both the adult and pediatric literature that information provided by proxy-respondents is not equivalent to that reported by the patient [5,6]. Imperfect agreement between self-report and proxy-report, termed cross-informant variance [7], has been consistently documented in the HRQOL measurement of children with chronic health conditions and healthy children [8-15]. However, even as pediatric patient self-report is advocated, there remains a fundamental role for parent proxy-report in pediatric clinical trials and health services research.

### The Role for parent proxy-report

While pediatric patient self-report should be considered the standard for measuring perceived HRQOL, there may be circumstances when the child is too young, too cognitively impaired, too ill or fatigued to complete a HRQOL instrument, and parent proxy-report may be needed in such cases [16]. Further, it is typically parents' perceptions of their children's HRQOL that influences healthcare utilization [17-19]. Thus, HRQOL instruments should be selected that measure the perspectives of both the child and parent since these perspectives may be independently related to healthcare utilization, risk factors, and quality of care [1].

In those cases in which pediatric patients are not able to provide self-report, reliable and valid parent proxy-report instruments are needed. For example, in a recent HRQOL study with pediatric patients with brain tumors, of those children ages 5–18 who were age eligible to self-report, 62% of the children were able to self-report [20]. Of the 99 families of children ages 2–18 who participated in this study of pediatric brain tumor patients, parent proxy-report was obtained from 99 parents, while pediatric patients who did not provide self-report included 17 patients who were toddlers (ages 2–4), 7 patients who reported they felt too ill to participate when approached, 11 who were determined to be cognitively delayed and unable to provide self-report, 5 who refused, and 4 who attempted to fill out the forms and became fatigued or became ill during the interview and were unable to finish during their clinic visit.

As is clear from this study and others in the literature, parent proxy-report instruments are required in situations such as this. Consequently, reliable and valid parent proxy-report instruments are essential primary outcome measures when children are unable to provide self-report. Ideally, parent and child HRQOL instruments should measure the same constructs with parallel items in order to make comparisons between self and proxy report more meaningful [21,22]. Even when children are able to self-report, parent proxy-report should be considered as a secondary outcome measure given parents' expanding role in clinical decision-making and home treatment regimens for pediatric chronic health conditions [1].

Recent FDA guidelines recommend that instrument development and validation testing for children and adolescents be conducted within fairly narrow age groupings and to determine the lower age limit at which reliable and valid responses can be compared across age categories [4]. Consistent with these recommendations, it has been an explicit goal of the PedsQL™ Measurement Model [9] to develop and test brief measures for the broadest age group empirically feasible [23,24]. The PedsQL™ 4.0 Generic Core Scales include child self-report for ages 5–18 and parent proxy-report for ages 2–18 [25,26]. The items chosen for inclusion were initially derived from the measurement properties of the child self-report scales, while the parent proxy-report scales were constructed to directly parallel the child self-report items.

Recently, we have reported on the feasibility, reliability, and validity of child self-report at the individual age subgroup level for ages 5–16 [27]. The objectives of the current analyses are to determine the feasibility, reliability and validity of parent proxy-report at the individual age subgroup level by parents of children 2–16 years of age utilizing data from the PedsQL™ 4.0 Generic Core Scales Database^SM ^on 13,878 children and adolescents. These analyses are consistent with the FDA guidelines recommending validation testing for children and adolescents within fairly narrow age groupings and the determination of the lower age limit for reliable and valid responses [4].

## Method

### Participants and settings

The sample contains parent proxy-report age subgroup data on 13,878 children ages 2 to 16 years from the PedsQL™ 4.0 Generic Core Scales Database^SM^(previously published data, n = 13,702, 98.7%; unpublished data, n = 176, 1.3%). Parents were recruited from general pediatric clinics, subspecialty clinics, and hospitals in which their children were being seen for well-child checks, mild acute illness, or chronic illness care (n = 3,718, 26.8%), and from a State Children's Health Insurance Program (SCHIP) in California (n = 10,160, 73.2%). Participants recruited from general pediatric clinics, subspecialty clinics, and hospitals were assessed in-person or by telephone. For in-person mode of administration, research assistants obtained written parental informed consent. Paper-and-pencil questionnaires were self-administered for parents and interview administered in situations in which the parent was unable to read or write. For telephone administration, parents of children ages 2 to 16 were called by a research assistant who explained the study, and obtained verbal parental informed consent. The research assistant verbally administered the PedsQL™ 4.0 to the parent. These research protocols were approved by the Institutional Review Board at Children's Hospital and Health Center, San Diego and other appropriate local Institutional Review Boards.

Parents recruited from the State Children's Health Insurance Program (SCHIP) were assessed via statewide mailing. PedsQL™ 4.0 paper-and-pencil surveys were mailed separately for each of the months of February and March 2001 to families with children ages 2–16 years throughout the State of California who were all new enrollees in SCHIP. Parents were instructed to complete the survey separately from their child, with the exception of parents of children ages 5 to 7 years, who were instructed to assist their child in completing the survey after completing their proxy-report. A reminder postcard followed the initial mailing, with a second survey mailed to nonrespondents. Nonrespondents to the second survey received a telephone reminder. Given that this project was conducted for program evaluation to comply with California Insurance Code 12693.92 (b), and not specifically research purposes, parents and children did not complete informed consent forms [28]. This protocol of analyzing existing deidentified data was approved by the Institutional Review Board at Children's Hospital and Health Center, San Diego.

For all forms combined (N = 13,878), the number of parent proxy respondents within each age subgroup is as follows: 1,309 two-year-olds (9.4%), 1,257 three-year-olds (9.1%), 1,198 four-year-olds (8.6%), 984 five-year-olds (7.1%), 1,134 six-year-olds (8.2%), 1,092 seven-year-olds (7.9%), 1,037 eight-year-olds (7.5%), 973 nine-year-olds (7.0%), 985 ten-year-olds (7.1%), 789 eleven-year-olds (5.7%), 774 twelve-year-olds (5.6%), 704 thirteen-year-olds (5.1%), 652 fourteen-year-olds (4.7%), 625 fifteen-year-olds (4.5%), and 365 sixteen-year-olds (2.6%). The sample contains parents of 7,143 boys (51.5%), 6,718 girls (48.4%), and 17 missing (0.1%). With respect to race/ethnicity, the sample consists of parents of a heterogeneous group of children with 7,395 Hispanics (53.3%), 3,011 White non-Hispanics (21.7%), 1,312 Asian or Pacific Islanders (9.5%), 585 Black non-Hispanics (4.2%), 70 American Indians or Alaskan Natives (0.5%), 177 other (1.3%), and 1,328 missing (9.6%). Parent surveys were completed in English (n = 7,497, 54.0%), Spanish (n = 5,713, 41.2%), Chinese (n = 325, 2.3%), Korean (n = 170, 1.2%), and Vietnamese (n = 141, 1.0%; missing = 32, 0.2%). Response equivalence has been previously demonstrated across language for the PedsQL™ by examining the percent missing data, floor and ceiling effects, and scale internal consistency across language, as well as across mode of administration [25].

The sample includes parents of healthy children, who were assessed either in physicians' offices during well-child checks and/or whose parents did not report the presence of a chronic health condition (n = 9,467, 68.2%), acutely ill children, whose parents did not report the presence of a chronic health condition, but who were assessed at one of the pediatric clinics or hospitals (n = 193, 1.4%), chronically ill children, whose parents reported the presence of a chronic health condition (i.e., a physical or mental health condition that has lasted or is expected to last at least 6 months and interferes with the child's activities) and/or were identified through their medical records as having a chronic health condition (n = 3,652, 26.3%), and 566 missing (4.1%). Within each age subgroup, the number of parent proxy-reports for healthy and chronically ill children is as follows: 1,069 healthy (81.7%) and 186 chronically ill (14.2%) two-year-olds, 946 healthy (75.3%) and 232 chronically ill (18.5%) three-year-olds, 907 healthy (75.7%) and 199 chronically ill (16.6%) four-year-olds, 693 healthy (70.4%) and 239 chronically ill (24.3%) five-year-olds, 851 healthy (75.0%) and 224 chronically ill (19.8%) six-year-olds, 774 healthy (70.9%) and 241 chronically ill (22.1%) seven-year-olds, 694 healthy (66.9%) and 296 chronically ill (28.5%) eight-year-olds, 651 healthy (66.9%) and 266 chronically ill (27.3%) nine-year-olds, 638 healthy (64.8%) and 305 chronically ill (31.0%) ten-year-olds, 491 healthy (62.2%) and 263 chronically ill (33.3%) eleven-year-olds, 482 healthy (62.3%) and 250 chronically ill (32.3%) twelve-year-olds, 398 healthy (56.5%) and 275 chronically ill (39.1%) thirteen-year-olds, 361 healthy (55.4%) and 255 chronically ill (39.1%) fourteen-year-olds, 347 healthy (55.5%) and 236 chronically ill (37.8%) fifteen-year-olds, and 165 healthy (45.2%) and 185 chronically ill (50.7%) sixteen-year-olds. Parent proxy-reports for the chronically ill sample (n = 3,652) are heterogeneous in terms of the children's diagnoses with 539 children diagnosed with cancer (14.8%), 520 children with asthma (14.2%), 314 with a cardiac condition (8.6%), 310 with diabetes (8.5%), 310 with a rheumatic condition (8.5%), 274 with a gastrointestinal condition (7.5%), 250 with cerebral palsy (6.8%), 108 with ADHD (3.0%), 103 with sickle cell anemia (2.8%), 100 diagnosed as obese (2.7%), 88 with renal disease (2.4%), 56 with mental health conditions (1.5%), and 680 with other chronic conditions (18.6%).

## Measures

### The PedsQL™ 4.0 (Pediatric Quality of Life Inventory™ Version 4.0)

The 23-item PedsQL™ 4.0 Generic Core Scales encompass: 1) Physical Functioning (8 items), 2) Emotional Functioning (5 items), 3) Social Functioning (5 items), and 4) School Functioning (5 items), and were developed through focus groups, cognitive interviews, pre-testing, and field testing measurement development protocols [9,25]. The instrument takes approximately 5 minutes to complete [25].

The PedsQL™ 4.0 Generic Core Scales are comprised of parallel child self-report and parent proxy-report formats. Child self-report includes ages 5–7, 8–12, and 13–18 years. Parent proxy-report includes ages 2–4 (toddler), 5–7 (young child), 8–12 (child), and 13–18 (adolescent), and assesses parent's perceptions of their child's HRQOL. The items for each of the forms are essentially identical, differing in developmentally appropriate language, or first or third person tense. The instructions ask how much of a problem each item has been during the past one month. A 5-point response scale is utilized across child self-report for ages 8–18 and parent proxy-report (0 = never a problem; 1 = almost never a problem; 2 = sometimes a problem; 3 = often a problem; 4 = almost always a problem).

Items are reverse-scored and linearly transformed to a 0–100 scale (0 = 100, 1 = 75, 2 = 50, 3 = 25, 4 = 0), so that higher scores indicate better HRQOL. Scale Scores are computed as the sum of the items divided by the number of items answered (this accounts for missing data). If more than 50% of the items in the scale are missing, the Scale Score is not computed. This accounts for the differences in sample sizes for scales reported in the Tables. Although there are other strategies for imputing missing values, this computation is consistent with the previous PedsQL™ peer-reviewed publications, as well as other well-established HRQOL measures [25,29,30]. For this study, over 98% of parent respondents were included in the Scale Score analyses after imputing missing values. The Physical Health Summary Score (8 items) is the same as the Physical Functioning Scale. To create the Psychosocial Health Summary Score (15 items), the mean is computed as the sum of the items divided by the number of items answered in the Emotional, Social, and School Functioning Scales.

### PedsQL™ Family Information Form

The PedsQL™ Family Information Form [25] or survey items adapted from the PedsQL™ Family Information Form were completed by parents. The PedsQL™ Family Information Form contains demographic information including the child's date of birth, gender, race/ethnicity, and parental education and occupation information required to calculate the Hollingshead socioeconomic status (SES) index [31]. One survey question asks the parent to report on the presence of a chronic health condition ("In the past 6 months, has your child had a chronic health condition?") defined as a physical or mental health condition that has lasted or is expected to last at least 6 months and interferes with the child's activities. If the parents check "Yes" to this question, they are asked to write in the name of the chronic health condition.

### Statistical analyses

The feasibility of parent proxy-report was determined from the percentage of missing values for the parent proxy-report sample as a whole and across each individual age subgroup from 2 to 16 years [29]. Items on the School Functioning Scale were excluded from the feasibility analysis for the 2 to 4 age subgroups given that toddlers do not necessarily attend school or daycare and thus, depending on whether their child was enrolled in school or daycare, parent proxy respondents were given the option of completing the School Functioning items. Scale internal consistency reliability was determined for parent proxy-report by calculating Cronbach's coefficient alpha across individual age subgroups [32]. Scales with reliabilities of 0.70 or greater are recommended for comparing patient groups, while a reliability criterion of 0.90 is recommended for analyzing individual patient scale scores [33,34]. Range of measurement was based on the percentage of scores at the extremes of the scaling range, that is, the maximum possible score (ceiling effect) and the minimum possible score (floor effect) [29].

Construct validity for parent proxy-report was determined utilizing the known-groups method. The known-groups method compares scale scores across groups known to differ in the health construct being investigated. In this study, PedsQL™ 4.0 Generic Core Scales Scores in groups differing in known health condition (healthy children and children known to have a chronic illness) were computed for the parent proxy-report sample across each age subgroup [29,35], using independent sample *t*-tests. In order to determine the magnitude of the anticipated differences, effect sizes were calculated [36]. Effect size as used in these analyses was calculated by taking the difference between the healthy sample mean and the chronic sample mean, divided by the healthy sample standard deviation. Effect sizes for differences in means are designated as small (0.20), medium (0.50), and large (0.80) in magnitude [36]. Statistical analyses were conducted using SPSS Version 13.0 for Windows.

## Results

### Feasibility

The percentage of missing item responses for the parent proxy-report sample as a whole was 2.1%. Items on the PedsQL™ 4.0 Generic Core Scales had minimal missing responses for parent proxy respondents across the age subgroups from 2 to 16 years. The percentage of missing item responses for parent proxy-report across the age subgroups was 2.2%, 1.9%, 1.9%, 3.4%, 2.2%, 2.2%, 2.1%, 1.8%, 1.9%, 1.9%, 2.0%, 1.2%, 2.0%, 2.0%, and 2.1% for age subgroups 2, 3, 4, 5, 6, 7, 8, 9, 10, 11, 12, 13, 14, 15, and 16, respectively. It should be noted that most of the missing data for ages 5–7 involved the School Functioning Scale (55.6%, 27.8%, and 30.0% for ages 5, 6, and 7, respectively). This is not a surprising finding, since young children do not necessarily attend school. When eliminating the School Functioning items, the percentage of missing items for the Total Scale Score is 1.9%, 1.6%, and 1.5%for ages 5, 6, and 7, respectively.

### Internal consistency reliability

Internal consistency reliability alpha coefficients for parent proxy-report across individual age subgroups are presented for the PedsQL™ 4.0 Generic Core Scales Total Scale Score in Table 1, Physical Health Summary Score in Table 2, Psychosocial Health Summary Score in Table 3, Emotional Functioning Scale Score in Table 4, Social Functioning Scale Score in Table 5, and School Functioning Scale Score in Table 6. Across the age subgroups, including for children as young as 2 years and as old as 16 years, the majority of the parent proxy-report scales exceed the minimum reliability standard of 0.70 required for group comparisons. The Total Scale Scores across the age subgroups approach or exceed the reliability criterion of 0.90 recommended for analyzing individual patient scale scores. Alpha values for parent proxy-report are lower for the School Functioning Scale Scores across the age subgroups, with the lowest alpha values on the School Functioning Scale for ages 2–4. Across the PedsQL™ scales and summary scores, internal consistency reliability alpha coefficients for parent proxy-report increase slightly with the child's age.

**Table 1:** PedsQL™ 4.0 Generic Core Scales Total Scale Score: Parent Proxy-Report Reliability and Validity

**Age**	**Reliability**		**Validity**									
			**Chronic Health Condition**				**Healthy Sample**					

	N	α	Mean	SD	% Floor	% Ceiling	Mean	SD	% Floor	% Ceiling	Chronic vs. Healthy Difference	Chronic vs. Healthy Effect Size

2 yrs	1,277	0.89	79.16	17.61	0.0	8.6	88.14	12.11	0.0	15.8	8.98*	0.74
3 yrs	1,232	0.90	76.90	18.15	0.0	5.7	87.96	11.63	0.0	13.4	11.06*	0.95
4 yrs	1,177	0.90	76.03	19.34	0.0	4.6	87.37	12.67	0.0	14.1	11.34*	0.90
5 yrs	893	0.92	70.57	19.19	0.4	1.6	79.91	15.85	0.0	7.6	9.34*	0.59
6 yrs	1,105	0.91	72.98	17.14	0.0	2.1	79.74	15.86	0.0	5.5	6.76*	0.43
7 yrs	1,061	0.92	70.04	19.12	0.0	2.7	78.59	16.53	0.0	8.0	8.55*	0.52
8 yrs	1,009	0.92	72.51	17.58	0.0	3.4	80.23	15.94	0.0	8.9	7.72*	0.48
9 yrs	952	0.92	69.34	18.32	0.4	0.7	78.89	17.07	0.0	7.5	9.55*	0.56
10 yrs	955	0.92	70.23	18.51	0.0	2.8	80.68	15.73	0.0	8.2	10.45*	0.66
11 yrs	768	0.93	71.26	18.71	0.0	2.1	80.95	16.39	0.0	9.4	9.69*	0.59
12 yrs	753	0.93	68.75	19.14	0.0	1.5	80.77	16.39	0.0	8.7	12.02*	0.73
13 yrs	692	0.93	71.11	18.24	0.0	1.4	80.24	17.02	0.0	9.8	9.13*	0.54
14 yrs	633	0.92	69.09	18.39	0.0	1.8	82.32	14.94	0.0	9.1	13.23*	0.89
15 yrs	605	0.93	71.20	17.48	0.4	1.2	80.67	16.42	0.0	12.7	9.47*	0.58
16 yrs	352	0.93	69.13	19.35	0.0	1.0	82.73	14.43	0.0	10.9	13.60*	0.94

Note: Total N = 13,464 for reliability, Total N = 13,028 for validity.

For the reliability analysis for the 2 to 4 age subgroups, items on the School Functioning Scale were excluded from the analysis given that toddlers do not necessarily attend school or daycare and thus, depending on whether their child was enrolled in school or daycare, parent proxy respondents were given the option of completing the School Functioning items.

Higher values equal better health-related quality of life.

% Floor/Ceiling = the percentage of scores at the extremes of the scaling range.

Effect sizes are designated as small (.20), medium (.50), and large (.80).

*p < .001 (independent samples t-test). f

**Table 2:** PedsQL™ 4.0 Generic Core Scales Physical Health Summary Score: Parent Proxy-Report Reliability and Validity

**Age**	**Reliability**		**Validity**									
			**Chronic Health Condition**				**Healthy Sample**					

	N	α	Mean	SD	% Floor	% Ceiling	Mean	SD	% Floor	% Ceiling	Chronic vs. Healthy Difference	Chronic vs. Healthy Effect Size

2 yrs	1,290	0.87	77.68	24.25	1.0	24.4	90.04	15.07	0.2	38.9	12.36*	0.82
3 yrs	1,238	0.87	76.17	25.05	0.8	19.3	90.07	14.24	0.0	37.6	13.90*	0.98
4 yrs	1,184	0.89	76.01	24.91	1.8	19.8	89.23	17.00	0.0	42.6	13.22*	0.78
5 yrs	975	0.88	68.79	26.36	1.2	11.3	79.94	20.64	0.0	23.8	11.15*	0.54
6 yrs	1,119	0.87	73.31	23.87	0.4	14.0	80.37	20.76	0.0	21.9	7.06*	0.34
7 yrs	1,084	0.88	70.88	26.23	1.1	14.4	79.52	21.35	0.0	23.3	8.64*	0.40
8 yrs	1,025	0.88	74.25	23.98	0.6	15.0	82.91	19.93	0.0	31.1	8.66*	0.43
9 yrs	963	0.89	70.47	24.86	1.8	12.6	81.52	21.85	0.0	32.0	11.05*	0.51
10 yrs	973	0.88	72.13	24.29	0.9	14.1	84.18	19.11	0.2	32.1	12.05*	0.63
11 yrs	783	0.90	73.29	25.16	1.4	12.8	82.66	20.95	0.2	31.2	9.37*	0.45
12 yrs	765	0.89	69.33	25.30	0.4	8.5	82.36	20.86	0.0	27.6	13.03*	0.62
13 yrs	701	0.89	71.50	23.63	1.4	12.0	82.37	21.67	0.0	32.2	10.87*	0.50
14 yrs	646	0.90	69.70	24.99	0.7	8.6	84.52	19.86	0.0	31.3	14.82*	0.75
15 yrs	617	0.88	72.95	22.16	0.4	8.4	82.43	20.48	0.0	30.0	9.48*	0.46
16 yrs	361	0.89	69.01	25.74	0.5	9.6	85.61	18.07	0.0	31.5	16.60*	0.92

Note: Total N = 13,724 for reliability, Total N = 13,004 for validity.

Higher values equal better health-related quality of life.

% Floor/Ceiling = the percentage of scores at the extremes of the scaling range.

Effect sizes are designated as small (.20), medium (.50), and large (.80).

*p < .001 (independent samples t-test).

**Table 3:** PedsQL™ 4.0 Generic Core Scales Psychosocial Health Summary Score: Parent Proxy-Report Reliability and Validity

**Age**	**Reliability**		**Validity**									
			**Chronic Health Condition**				**Healthy Sample**					

	N	α	Mean	SD	% Floor	% Ceiling	Mean	SD	% Floor	% Ceiling	Chronic vs. Healthy Difference	Chronic vs. Healthy Effect Size

2 yrs	1,282	0.81	79.90	15.34	0.0	10.7	86.83	12.41	0.0	19.6	6.93*	0.56
3 yrs	1,239	0.81	77.32	16.29	0.0	7.8	86.41	12.16	0.1	16.0	9.09*	0.75
4 yrs	1,178	0.82	75.98	17.79	0.0	6.9	86.17	12.34	0.0	16.1	10.19*	0.83
5 yrs	897	0.87	71.66	18.30	0.4	2.3	79.87	15.15	0.0	8.8	8.21*	0.54
6 yrs	1,111	0.86	73.02	16.49	0.0	3.7	79.37	15.26	0.0	7.3	6.35*	0.42
7 yrs	1,063	0.88	69.61	18.56	0.0	3.0	78.08	16.11	0.0	8.9	8.47*	0.53
8 yrs	1,011	0.87	71.66	17.10	0.0	3.7	78.82	16.04	0.0	10.4	7.16*	0.45
9 yrs	958	0.87	68.98	17.68	0.4	0.7	77.39	16.81	0.0	9.2	8.41*	0.50
10 yrs	964	0.88	69.35	18.72	0.0	3.4	78.83	16.14	0.0	9.6	9.48*	0.59
11 yrs	769	0.88	70.18	18.13	0.0	3.2	80.00	16.22	0.0	11.0	9.82*	0.61
12 yrs	757	0.90	68.51	18.28	0.0	1.8	79.86	16.18	0.0	10.8	11.35*	0.70
13 yrs	693	0.89	70.91	18.45	0.0	2.4	79.07	17.18	0.0	11.3	8.16*	0.47
14 yrs	633	0.89	68.76	18.35	0.0	3.9	81.08	15.11	0.0	11.4	12.32*	0.82
15 yrs	611	0.89	70.22	17.85	0.4	1.2	79.62	16.51	0.0	15.9	9.40*	0.57
16 yrs	354	0.89	68.98	19.38	0.0	3.5	81.23	14.75	0.0	11.5	12.25*	0.83

Note: Total N = 13,520 for reliability, Total N = 13,019 for validity.

For the reliability analysis for the 2 to 4 age subgroups, items on the School Functioning Scale were excluded from the analysis given that toddlers do not necessarily attend school or daycare and thus, depending on whether their child was enrolled in school or daycare, parent proxy respondents were given the option of completing the School Functioning items. Higher values equal better health-related quality of life.

% Floor/Ceiling = the percentage of scores at the extremes of the scaling range.

Effect sizes are designated as small (.20), medium (.50), and large (.80).

*p < .001 (independent samples t-test).

**Table 4:** PedsQL™ 4.0 Generic Core Scales Emotional Functioning Scale Score: Parent Proxy-Report Reliability and Validity

**Age**	**Reliability**		**Validity**									
			**Chronic Health Condition**				**Healthy Sample**					

	N	α	Mean	SD	% Floor	% Ceiling	Mean	SD	% Floor	% Ceiling	Chronic vs. Healthy Difference	Chronic vs. Healthy Effect Size

2 yrs	1,290	0.75	75.38	18.46	0.5	15.7	84.55	14.00	0.0	25.4	9.17*	0.66
3 yrs	1,242	0.77	75.28	18.07	0.0	14.8	83.48	14.52	0.1	22.2	8.20*	0.56
4 yrs	1,181	0.78	73.17	19.70	0.0	12.4	84.72	13.95	0.0	25.8	11.55*	0.83
5 yrs	976	0.79	71.17	20.59	0.8	10.5	79.76	15.88	0.1	17.9	8.59*	0.54
6 yrs	1,123	0.78	73.41	19.86	0.8	16.5	80.11	15.94	0.0	19.0	6.70*	0.42
7 yrs	1,082	0.81	69.74	21.77	0.4	13.3	79.64	16.85	0.1	21.1	9.90*	0.59
8 yrs	1,025	0.81	69.95	20.47	0.3	12.6	78.98	17.94	0.1	22.9	9.03*	0.50
9 yrs	968	0.81	69.10	20.35	0.7	9.4	78.88	17.98	0.3	19.8	9.78*	0.54
10 yrs	979	0.82	69.26	20.95	0.3	12.9	79.48	18.00	0.3	23.7	10.22*	0.57
11 yrs	784	0.83	67.42	21.88	0.7	8.2	80.46	18.02	0.0	24.8	13.04*	0.72
12 yrs	769	0.83	68.34	20.78	0.0	9.6	79.63	17.86	0.4	22.2	11.29*	0.63
13 yrs	702	0.84	68.43	21.39	0.7	10.7	80.32	18.39	0.0	24.1	11.89*	0.65
14 yrs	646	0.81	67.03	21.61	0.4	11.8	80.43	16.95	0.0	23.5	13.40*	0.79
15 yrs	620	0.85	69.31	21.96	0.8	10.0	79.14	18.86	0.3	26.8	9.83*	0.52
16 yrs	363	0.85	66.25	22.54	0.5	10.6	80.89	18.04	0.0	21.2	14.64*	0.81

Note: Total N = 13,750 for reliability, Total N = 13,004 for validity.

Higher values equal better health-related quality of life.

% Floor/Ceiling = the percentage of scores at the extremes of the scaling range.

Effect sizes are designated as small (.20), medium (.50), and large (.80).

*p < .001 (independent samples t-test).

**Table 5:** PedsQL™ 4.0 Generic Core Scales Social Functioning Scale Score: Parent Proxy-Report Reliability and Validity

**Age**	**Reliability**		**Validity**									
			**Chronic Health Condition**				**Healthy Sample**					

	N	α	Mean	SD	% Floor	% Ceiling	Mean	SD	% Floor	% Ceiling	Chronic vs. Healthy Difference	Chronic vs. Healthy Effect Size

2 yrs	1,288	0.78	84.03	19.78	0.0	34.5	88.55	16.08	0.2	46.5	4.52**	0.28
3 yrs	1,240	0.74	81.08	19.89	0.0	28.3	88.89	14.13	0.1	42.3	7.81***	0.55
4 yrs	1,182	0.78	79.76	20.61	0.5	27.2	87.75	16.65	0.2	44.7	7.99***	0.48
5 yrs	974	0.77	74.28	21.33	0.0	19.8	81.03	20.23	0.3	32.2	6.75***	0.33
6 yrs	1,120	0.76	74.87	21.68	0.0	19.8	80.58	20.42	0.1	28.8	5.71***	0.28
7 yrs	1,083	0.78	72.73	22.49	0.0	16.0	78.99	21.30	0.1	29.7	6.26***	0.29
8 yrs	1,026	0.77	77.05	20.72	0.0	23.3	80.70	20.93	0.3	34.3	3.65*	0.17
9 yrs	968	0.79	72.58	22.44	0.4	15.5	78.13	23.03	0.5	31.8	5.55**	0.24
10 yrs	979	0.80	73.14	23.69	0.3	19.7	81.41	20.68	0.0	33.5	8.27***	0.40
11 yrs	779	0.78	76.19	21.27	0.7	19.9	82.98	20.07	0.2	37.3	6.79***	0.34
12 yrs	768	0.80	72.09	22.68	0.0	19.9	83.03	19.63	0.0	36.9	10.94***	0.56
13 yrs	699	0.82	77.14	23.19	0.3	25.4	81.39	20.95	0.3	38.9	4.25*	0.20
14 yrs	646	0.82	73.43	23.41	0.4	21.4	85.32	19.03	0.0	46.0	11.89***	0.62
15 yrs	623	0.81	76.82	22.46	0.4	25.7	83.33	19.50	0.3	42.4	6.51***	0.33
16 yrs	361	0.81	75.01	23.59	1.0	21.7	85.40	17.91	0.0	42.4	10.39***	0.58

Note: Total N = 13,736 for reliability, Total N = 12,991 for validity.

Higher values equal better health-related quality of life.

% Floor/Ceiling = the percentage of scores at the extremes of the scaling range.

Effect sizes are designated as small (.20), medium (.50), and large (.80).

*p < .05, **p < .01, ***p < .001 (independent samples t-test).

**Table 6:** PedsQL™ 4.0 Generic Core Scales School Functioning Scale Score: Parent Proxy-Report Reliability and Validity

**Age**	**Reliability**		**Validity**									
			**Chronic Health Condition**				**Healthy Sample**					

	N	α	Mean	SD	% Floor	% Ceiling	Mean	SD	% Floor	% Ceiling	Chronic vs. Healthy Difference	Chronic vs. Healthy Effect Size

2 yrs	438	0.59	82.08	19.52	0.0	13.7	89.89	14.95	0.0	19.5	7.81*	0.52
3 yrs	592	0.69	70.71	24.41	0.8	10.7	88.31	16.05	0.1	23.4	17.60*	1.10
4 yrs	798	0.61	72.72	24.94	1.4	13.8	86.18	16.60	0.0	30.1	13.46*	0.81
5 yrs	908	0.74	69.04	22.62	1.2	10.5	78.47	18.93	0.0	17.6	9.43*	0.50
6 yrs	1,117	0.72	70.75	19.44	0.0	7.4	77.36	19.45	0.0	17.6	6.61*	0.34
7 yrs	1,068	0.74	66.23	22.64	1.1	7.2	75.65	19.95	0.1	17.1	9.42*	0.47
8 yrs	1,019	0.74	68.31	21.16	0.9	7.4	76.59	19.22	0.1	18.4	8.28*	0.43
9 yrs	962	0.73	65.31	21.68	0.4	5.8	74.98	20.02	0.0	16.9	9.67*	0.48
10 yrs	970	0.77	66.10	22.96	0.6	9.1	75.42	20.03	0.2	17.7	9.32*	0.47
11 yrs	774	0.77	67.02	23.06	0.4	6.0	76.65	20.16	0.2	20.6	9.63*	0.48
12 yrs	761	0.78	65.17	21.95	0.7	7.4	76.98	19.48	0.0	18.7	11.81*	0.61
13 yrs	699	0.76	67.22	21.91	0.0	9.6	75.31	21.13	0.0	20.6	8.09*	0.38
14 yrs	638	0.78	65.93	21.34	0.0	7.5	77.54	19.82	0.0	23.8	11.61*	0.59
15 yrs	613	0.79	64.53	21.69	0.8	6.4	76.25	20.42	0.0	22.8	11.72*	0.57
16 yrs	355	0.79	65.70	23.17	0.5	9.1	77.26	18.83	0.0	20.6	11.56*	0.61

Note: Total N = 11,712 for reliability, Total N = 11,106 for validity.

Higher values equal better health-related quality of life.

% Floor/Ceiling = the percentage of scores at the extremes of the scaling range.

Effect sizes are designated as small (.20), medium (.50), and large (.80).

*p < .001 (independent samples t-test).

### Range of measurement

Tables 1 through 6 present the percentages of parent proxy-reported scores at the floor and ceiling for healthy children and children with a known chronic health condition across the age subgroups. There were minimal floor effects for the healthy and chronic health condition samples across the age subgroups, with the majority of scales demonstrating less than 1.0% of respondents scoring at the minimum. In some cases, ceiling effects existed. These ranged from minimal (e.g., 5.5% of respondents in the 6 year old healthy subgroup for the proxy-report Total Scale Score) to moderate (e.g., 46.5% of respondents in the 2 year old subgroup for the proxy-report Social Functioning Scale). The ceiling effects were in the expected direction, with parents of healthy children reporting more ceiling effects than parents of children with a known chronic health condition.

### Construct validity

Tables 1 through 6 demonstrate comparisons between parent proxy-reported PedsQL™ 4.0 Generic Core Scales Total Scale Scores, Physical Health Summary Scores, Psychosocial Health Summary Scores, Emotional Functioning Scale Scores, Social Functioning Scale Scores, and School Functioning Scale Scores for healthy children and children with a known chronic health condition by individual age subgroups. For each PedsQL™ scale and summary score, across the age subgroups, including children as young as 2 years and as old as 16 years, parent proxy-report for healthy children demonstrated a statistically significant difference in HRQOL (better HRQOL) than parent proxy-report for children with a known chronic health condition, with most effect sizes in the medium to large effect size range [36].

### Parent/Child agreement

Agreement between child self-report and parent proxy-report on the PedsQL™ 4.0 Generic Core Scales has been previously reported for individual age subgroups from 5 to 16 years [27]. Two-way mixed effect model (absolute agreement, single measure) Intraclass Correlations (ICC) were designated as ≤ 0.40 poor to fair agreement, 0.41–0.60 moderate agreement, 0.61–0.80 good agreement, and 0.81–1.00 excellent agreement [37,38]. Moderate to good agreement was found across most of the PedsQL™ scales and summary scores. The average ICCs across the individual age subgroups from 5 to 16 years for the PedsQL™ Total Scale Score, Physical Health Summary Score, Psychosocial Health Summary Score, Emotional Functioning Scale Score, Social Functioning Scale Score, and School Functioning Scale Score were 0.60, 0.49, 0.63, 0.64, 0.52, and 0.55, respectively.

## Discussion

The results demonstrate the feasibility, reliability, and validity of parent proxy-report at individual age subgroups for ages 2–16 years. It should be noted that even though the 2–4 age subgroups had the lowest coefficient alpha reliability coefficients for the School Functioning Scale, this scale is reworded and simplified to a 3-item scale, rather than the 5-item scale used for ages 5–18, which may attenuate the achievable reliability coefficients relative to the 5-item scales [33]. This may explain in part the lower reliability coefficients for the 2–4 age subgroups in comparison to the 5–16 age subgroups for the School Functioning Scale. The relatively large number of missing data for the School Functioning Scale for the 2–4 subgroups may have further attenuated the potentially achievable reliability coefficients for these age subgroups [33]. Although Cronbach alpha represents the lower bound of the reliability of a measurement instrument, and is a conservative estimate of actual reliability [39], scales that do not approach or meet the 0.70 standard should be used only for descriptive analyses. Finally, research on the factors which may influence the level of agreement between pediatric patients and their parents is emerging, with age and health status as potential factors among others [40]. Identifying the conditions under which parent proxy-report instruments achieve better agreement with child self-report instruments will facilitate the interpretation of HRQOL outcomes across clinical trials when child self-report is not attainable. A research strategy in which pediatric patient self-report instruments are utilized as primary outcome measures, while parent proxy-report instruments serve as secondary outcome measures, would further enable comparisons across clinical trials.

## Conclusion

While pediatric patient self-report should be considered the standard for measuring perceived HRQOL, there are circumstances when children are too young, too cognitively impaired, too ill or fatigued to complete a HRQOL instrument, and reliable and valid parent proxy-report instruments are needed. Parent and child HRQOL instruments should measure the same constructs with parallel items in order to make comparisons between self and proxy report more meaningful, with demonstrated feasibility, reliability, and validity at individual age subgroups. This level of individual age subgroup analysis is consistent with recent FDA guidelines. Measuring health from the perspective of children and their parents provides a level of accountability consistent with the Institute of Medicine report on the quality of care [41]. As the consumers of pediatric healthcare, families are uniquely positioned to give their perspectives on healthcare quality through their perceptions of pediatric health-related quality of life.

## Abbreviations

HRQOL Health-Related Quality of Life

PedsQL™ Pediatric Quality of Life Inventory™

PRO Patient-Reported Outcomes

FDA Food and Drug Administration

## Competing interests

Dr. Varni holds the copyright and the trademark for the PedsQL™ and receives financial compensation from the Mapi Research Trust, which is a nonprofit research institute that charges distribution fees to for-profit companies that use the Pediatric Quality of Life Inventory™. The PedsQL™ is available at the PedsQL™ Website [42].

## Authors' contributions

JWV conceptualized the rationale and design of the study. JWV and CAL drafted the manuscript. CAL performed the statistical analyses. TMB participated in the statistical analyses. All authors read and approved the final manuscript.
